# Referral-based transition to subsequent rehabilitation at home after stroke: one-year outcomes and use of healthcare services

**DOI:** 10.1186/s12913-022-08000-7

**Published:** 2022-05-03

**Authors:** Sebastian Lindblom, Malin Tistad, Maria Flink, Ann Charlotte Laska, Lena von Koch, Charlotte Ytterberg

**Affiliations:** 1 Department of Neurobiology, Care Sciences and Society, Karolinska Institutet, Huddinge, Sweden; 2 Karolinska University Hospital, Stockholm, Sweden; 3 Dalarna University, School of Health and Social Studies, Falun, Sweden; 4 Karolinska Institutet, Department of Clinical Sciences Danderyd Hospital, Stockholm, Sweden

**Keywords:** Healthcare use, Rehabilitation, Care transition, Home environment, Readmission primary care, Hospitalization, Patient discharge

## Abstract

**Background:**

There is a lack of knowledge about patients’ journeys across the stroke care continuum, especially regarding the transition from inpatient to outpatient care and rehabilitation. Therefore, the aim of the present study was to explore and describe patterns of healthcare use over a one-year period, health outcomes at 3 and 12 months for patients following a referral-based transition to subsequent rehabilitation in the home, and the caregiver burden on their significant others. A further aim was to explore factors associated with the use of rehabilitation and healthcare after the referral-based transition to continued rehabilitation in the home for people recovering from a stroke.

**Methods:**

Data regarding healthcare use during the first 12 months post-stroke was collected from the Region Stockholm computerized register. Data on patient characteristics, disease-related data, and functioning were retrieved drawn from medical records and questionnaires. Descriptive statistics were used to present healthcare use, participants’ characteristics, disease-related data, and patient functioning. Multivariable regression models were created to explore associations between the total number of outpatient contacts, total visits with the neurorehabilitation team, and the independent variables.

**Results:**

The mean age for the 190 participants was 73 years for men and 78 years for women. Twenty-one participants (11%) had an acute rehospitalization within 30 days after discharge, and 41 participants (21%) were re-hospitalized within 90 days. Twenty-two (12%) of the participants had no visits with the neurorehabilitation team, 73 (39%) participants had 1–3 visits, 57 (30%) had 4–16 visits, and 38 (20%) had ≥17 visits. Female sex and length of hospital stay were associated with a higher number of visits with the neurorehabilitation team. Living alone, higher self-rated recovery, and being able to walk independently were associated with a lower number of visits with the neurorehabilitation team. Female sex, having home help services before the stroke, longer length of hospital stay, and more comorbidities were associated with a higher number of outpatient contacts.

**Conclusions:**

The findings indicate that there is no generic pattern of healthcare use during the first-year post-stroke in patients receiving referral-based transition to continued rehabilitation in the home. The different patterns of healthcare use seemed to mirror the participants’ level of functioning. However, there is a need to further investigate how follow-up and rehabilitation correspond to the needs of patients and their significant others in the short- and long-term perspective.

**Trial registration:**

ClinicalTrials.gov, registration number: NCT02925871. Date of registration: October 6, 2016.

## Background

Stroke is the second-leading cause of death [1] and disability [2] worldwide. Over 13 million people suffer a stroke each year, and the overall stroke burden entails serious social and economic consequences for society [3, 4]. In order to address these consequences, efforts need to be made to ensure a high-quality healthcare system that integrates and coordinates the different phases of the stroke care continuum: prehospital and emergency care, acute inpatient care, rehabilitation, and community reintegration [5]. The challenges for patients with stroke are often related to the gaps between these phases and the different levels of care, especially the transition from inpatient care to outpatient care and rehabilitation [6–11].

Following the sudden onset of stroke, prehospital and emergency care followed by acute inpatient stroke care is warranted. Strong evidence supports the provision of such care in specialized stroke units [12]. In Sweden, about 90% of the stroke population patients are treated in stroke units during the acute phase. There are large regional variations in the organization of care and rehabilitation after discharge from the stroke unit. Approximately 50% are discharged directly to home with or without subsequent rehabilitation and the remaining to inpatient rehabilitation, short-term accommodation, or nursing homes [13].

Improvements in acute medical treatment, together with limited access to available beds in acute stroke units and geriatric wards, have contributed to a decreased length of hospital stays and early discharge from the hospital [13, 14]. The short length of inpatient stays and the limited time for rehabilitation and preparation for self-management before discharge generates a need for a period of rehabilitation after discharge to recover and regain function. Most recovery of impairments after stroke is proposed to take place during the first 6 months [15]. However, there is a lack of knowledge about patients’ journeys across the transitions in care to continued rehabilitation and about the stroke continuum as a whole [16].

One health service model that bridges inpatient care with continued rehabilitation in the home, for which there is strong evidence of beneficial effects, is Early Supported Discharge (ESD) [17]. The health service includes a supported discharge process in which an interdisciplinary team plans and coordinates the discharge together with the patient and continues the subsequent rehabilitation in the home environment [18]. Nevertheless, in the context of a complex health care system, ESD has proven to be difficult to implement in clinical practice [13]. Instead, services with some similarities to ESD have been implemented, such as health services with electronic referral to subsequent rehabilitation in the home, as in the Stockholm region, in Sweden. The experiences of patients, significant others, and professionals with referral-based transition (RBT) to subsequent rehabilitation at home have been explored in previous studies, suggesting a need for improvements regarding cross-organizational dialogue and increased involvement of patients and significant others [7]. However, there is a lack of knowledge regarding the extent and when in time during the first year after stroke patients receive and use subsequent rehabilitation at home and other healthcare services after RBT, as well as outcomes for patients and significant others. Furthermore, factors associated with the use of rehabilitation and healthcare after RBT are unknown.

Therefore, the aim of the present study was to explore and describe patterns of healthcare use over a one-year period and health outcomes at 3 and 12 months for patients receiving RBT to subsequent rehabilitation at home, and the caregiver burden on their significant others. A further aim was to explore factors associated with the use of rehabilitation and healthcare after RBT to continued rehabilitation in the home for people with stroke.

## Methods

### Study design

Prospective observational study.

### Study context

The study was conducted in the Stockholm region in Sweden. Similar to healthcare services in Sweden in general, rehabilitation is publicly funded. At the onset of stroke, people are admitted to acute hospital care and rehabilitation at a stroke unit. Since 2012, RBT to continued rehabilitation in the home environment has been offered to those who are discharged to their home after a stroke. The subsequent rehabilitation in the home is supplied by a neurorehabilitation team organized through the primary healthcare system. The team consists of allied health professionals such as physiotherapists, occupational therapists, speech and language therapists, dieticians, and social workers. The transition is initiated by an electronic referral from the hospital staff to the receiving neurorehabilitation team. The referral notifies the neurorehabilitation team about the patient and their discharge. The neurorehabilitation team is obligated to initiate contact within 48 hours after the referral and/or hospital discharge. The operation of the neurorehabilitation team is not constrained by any demands or limitations regarding the number of visits or duration of treatment.

### Participants and procedures

Patients with a suspected stroke who received RBT to continued rehabilitation in the home from either the acute stroke unit or the geriatric stroke ward at two different hospitals in the Stockholm region between April 2016 and February 2018 were eligible for inclusion. All patients initially received care at the acute stroke unit and before discharged home some were referred to the geriatric stroke ward.

Participants were recruited by the allied healthcare professionals at the hospitals: the physiotherapists, occupational therapists, speech and language therapists, and social workers who were responsible for sending the electronic referral to the neurorehabilitation teams. Eligible patients received oral and written information about the study. The included patients were asked if they were willing to identify a significant other e.g., a partner, child, or friend who would consider participating in the study. Written informed consent was obtained from patients and the significant others who agreed to participate. At 3 and 12 months, a structured face-to-face interview involving questionnaires and performance-based tests was conducted during a home visit. The study was approved by the Swedish Ethical Review Authority, Regional Ethics Committee in Stockholm, approval number: 2015/1923–31/2, and procedures were conducted in accordance with the Declaration of Helsinki.

### Data collection

#### Healthcare use

Data regarding the use of healthcare services during the first 12 months post-stroke was collected from the Stockholm Region computerized register. Inpatient care was categorized as the initial hospitalization length of stay, and recurrent hospitalizations as the number of hospitalizations, length of stay, and the number of days after initial discharge post-stroke. Outpatient contacts included contacts with specialist care, emergency visits, primary care, home care, rehabilitation with the neurorehabilitation team, other types of rehabilitation, and other outpatient contacts, such as laboratory testing, radiology, and mammography. These outpatient contacts were further categorized based on profession and type of contact: i.e., home visit, clinic visit, or remote contact.

#### Patient characteristics

Patient characteristics were retrieved from the medical records and comprised age, sex, civil status (living alone or cohabiting), educational level (elementary, secondary, or college/university), work status (working or not working), and use of home help services before the stroke (yes or no).

#### Disease-related data

Disease-related data comprised type of stroke (ischemic or intracerebral hemorrhage), aphasia (yes/no), reperfusion therapy (yes/no), stroke severity, and comorbidity. Stroke severity was categorized based on scores on the Barthel Index (BI) [19, 20] and was collected from medical records before discharge and at the 3- and 12-month follow-up visits. The BI contains ten variables on personal activities of daily living (ADL) and mobility that are scored as 0, 5, or 10 points, generating a sum total between 0 and 100, where a higher score indicates a higher degree of independence. As the BI has shown good agreement with other stroke severity measures, the BI at baseline was categorized as moderate/severe stroke (0–49), mild stroke (50–94), or very mild stroke (95–100) [21]. Data on comorbidities were collected from the medical records and categorized using the Charlson Comorbidity Index (CCI), consisting of 19 conditions weighted from 1 to 6 according to the 1-year mortality risk [22]. The CCI was categorized as no comorbidity (score of 0), low comorbidity (score of 1–2), and moderate/severe comorbidity (score of > 2).

Perceived recovery from stroke was collected before discharge and at 3 and 12 months. The participants rated their perceived recovery from the stroke using a sub-scale of the Stroke impact scale [23, 24]. The sub-scale uses a visual analog scale from 0 to 100, where 0 indicates “not recovered at all” and 100 indicates “fully recovered” after a stroke.

#### Functioning

Data on functioning comprised ADL, walking ability, and self-rated recovery. ADL pre- and post-stroke was assessed using the KATZ Personal-ADL (PADL) Index [25, 26] collected from medical records and at the 3- and 12-month home visits. The index consists of six activities, each being given a score of 1 if the person is able to do it independently and 0 if the person is dependent, resulting in a total score from 0 to 6. A score of ≤5 was categorized as dependent on the PADL and > 5 as independent. Instrumental ADL (IADL) was assessed using the KATZ IADL Index and was collected at the 3- and 12-month home visits [25, 26]. The index consists of four activities: cooking, cleaning, transportation, and shopping. Each item is given a score of 1 if the person is able to do it independently and 0 if the person is dependent in the activity, resulting in a total score of 0 to 4. Data on walking ability were collected from medical records and the 3- and 12-month home visits and was categorized as unable to walk/walks with assistance and support, walks with a walking aid, or walks without aid or support.

#### Burden on significant others

The Caregiver Burden Scale (CBS) was used to describe the subjective burden on significant others [27]. The scale consists of 22 items scored on a 4-point Likert scale (1–4) related to the significant other’s health, psychological well-being, relationships, social network, physical workload, and environmental aspects. The total score ranges from 22 to 88 points, with higher scores indicating a higher burden on the significant other.

### Statistical analysis

Descriptive statistics were used to present healthcare use, characteristics of patients and significant others, disease-related data, patient functioning, and burden on significant others. The mean and standard deviation were used to describe normally distributed data, and the median and interquartile range (IQR) were used to describe non-normally distributed data. The extent of visits and time period for visits with the neurorehabilitation team was the point of departure for identification of patterns of healthcare use: no visits; follow-ups only i.e., 1–3 visits; > 3 visits; 4–16 visits during < 6 months; and > 17 visits during > 6 months. Two different multivariable regression models that included the whole sample were created to explore associations between a) total visits with the neurorehabilitation team (dependent variable) and b) total outpatient contacts (dependent variable) and the independent variables. In both models, the independent variables at baseline were age, sex, civil status, educational level, work status, use of home help services before stroke, reperfusion therapy, stroke severity, comorbidity, PADL, walking ability, self-rated recovery, length of hospital stay during initial hospitalization and number of total outpatient contacts or total neurorehabilitation contacts when they were not used as the dependent variables. Since both dependent variables were skewed, they were log-transformed.

## Results

**Fig. 1:**
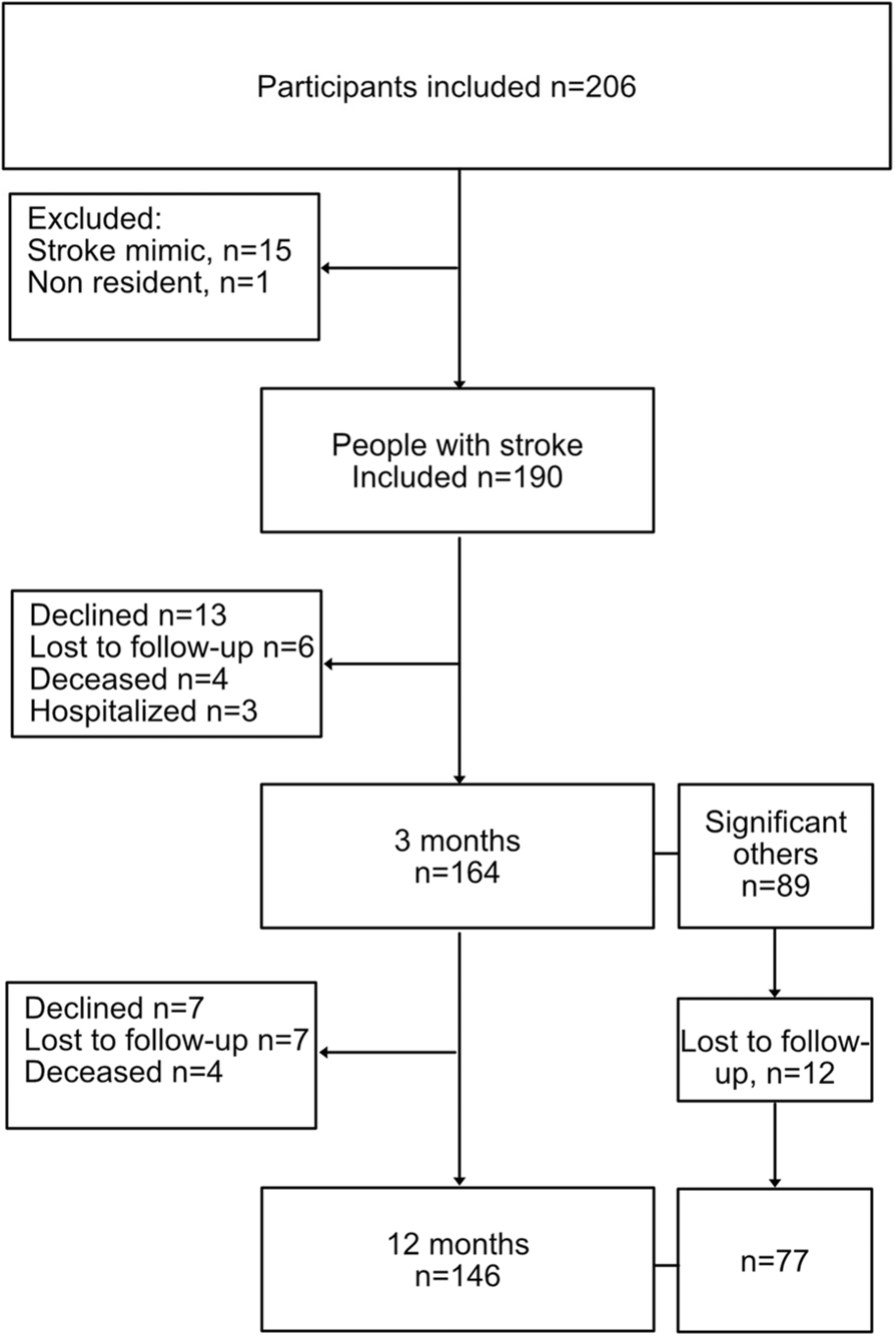
Flowchart of the inclusion and retention of people with stroke and significant others

In total, 206 participants were included in the study. Sixteen participants were excluded due to either stroke mimic (*n* = 15) or not being a resident of the Stockholm region (*n* = 1), leaving 190 people included in the analysis. In total, 89 significant others were included in the study. A flowchart of the inclusion and retention of participants can be seen in Fig. 1. The patient characteristics, disease-related data, and functioning at the time of discharge are presented in Table 1. The mean age for men was 73, and for women it was 78 years. The characteristics of the significant others can be seen in Table 2. Of the 89 participating significant others, 25 were men, with a mean age of 67 years, and 64 were women, with a mean age of 68 years. Four different patterns of visits with the neurorehabilitation team were identified. Twenty-two participants had no visits with the neurorehabilitation team (pattern 1), 73 participants had 1–3 visits (pattern 2), 57 had 4–16 visits during the first two quarters (pattern 3), and 38 had more than 17 visits during more than the first two quarters (pattern 4) in relation to the stroke for which they were included in the study. Out of the 22 participants in pattern 1 who had no visits with the neurorehabilitation team, 18 had declined a visit, and for the remaining 4 no explanatory reason was found. Individuals with moderate/severe stroke were represented in all patterns, but the median score for BI at the time of discharge was 95 for patients in patterns 1 and 2, 90 for patients in pattern 3, and 85 for patients in pattern 4. Recurrent hospitalization occurred within all patterns. The median total length of stay in the hospital was 3 days for patients in patterns 1 and 2, 11 days for patients in pattern 3, and 17 days for patients in pattern 4. Twenty-one participants (11%) had an acute hospitalization within 30 days of discharge, and 41 participants (21%) had a hospitalization within 90 days. During the 12-month study period, 18 participants (9%) had a recurrent stroke or transient ischemic attack.

**Table 1:** Patient characteristics, disease-related data, and functioning at the time of discharge

Variable	Pattern 1 ***n*** = 22	Pattern 2 ***n*** = 73	Pattern 3 ***n*** = 57	Pattern 4 ***n*** = 38	Total ***n*** = 190
Age, mean (SD), min-max	70 (15) 39–93	75 (12) 39–97	77 (13) 35–99	76 (10) 45–91	75 (12) 35–99
Sex, male, n (%)	16 (71)	49 (67)	27 (47)	20 (53)	112 (59)
Cohabiting, n (%)	15 (68)	49 (67)	34 (60)	25 (66)	123 (65)
Education, n (%)					
Elementary/Secondary	13 (59)^a^	41 (56)^c^	32 (56)^c^	21 (55)^a^	107 (56)^d^
University	8 (36)	27 (37)	20 (35)	16 (42)	71 (37)
Working, n (%)	6 (27)	17 (23)	9 (16)	5 (13)	37 (20)
Help from home services before stroke, n (%)	3 (14)	12 (16)	15 (26)	10 (26)	40 (21)
Type of stroke, n (%)					
Ischemic	21 (96)	70 (96)	53 (93)	33 (87)	177 (93)
Intracerebral hemorrhage	1 (4)	3 (4)	4 (7)	5 (13)	13 (7)
Aphasia, n (%)	3 (14)	7 (10)	5 (9)	6 (16)	21 (11)
Reperfusion therapy, n (%)	5 (23)	17 (23)	9 (16)	4 (11)	35 (18)
Stroke severity, n (%)					
Very mild	17 (77)	49 (67)	23 (40)	11 (29)	100 (53)
Mild	4 (18)	22 (30)	30 (53)	19 (50)	75 (39)
Moderate/severe	1 (5)	2 (3)	4 (7)	8 (21)	15 (8)
Charlson Index, n (%)					
No comorbidity	11 (50)	41 (56)	24 (42)	17 (45)	93 (49)
Low comorbidity	8 (36)	25 (34)	23 (40)	15 (39)	71 (37)
Moderate/severe comorbidity	3 (14)	7 (10)	10 (18)	6 (16)	26 (14)
Katz ADL before stroke, dependent, n (%)	2 (9)	3 (4)	9 (16)	6 (16)	20 (10)
Katz ADL post-stroke, dependent, n (%)	3 (14)	14 (19)	25 (44)	22 (58)	64 (34)
Barthel Index, median (IQR) min-max	95 (94–100) 5–100	95 (90–100) 30–100	90 (78–100) 0–100	85 (60–95) 15–100	95 (85–100) 0–100
Walking ability, n (%)					
Walks independently without aid and support	18 (82)	49 (67)	20 (35)	7 (18)	94 (50)
Walks with walking aid	2 (9)	18 (25)	25 (44)	14 (37)	59 (31)
Unable to walk/walks with assistance and support	2 (9)	6 (8)	12 (21)	17 (45)	37 (19)
SIS Recovery, mean (SD) min-max	64 (36) 0–100	67 (27) 0-100^a^	61 (21) 10–100	46 (25) 0-100^a^	62 (46–80) 0-100^b^
Length of stay, days, median (IQR) min-max					
Stroke unit	3 (2–3) 1–6	3 (2–4) 1–11	4 (2–6) 1–25	4 (2–6) 0–28	3 (2–5) 0–28
Geriatric ward^e^	16 (13–19) 13–19	9 (8–13) 1–15	14 (8–17) 7–54	15 (11–22) 5–80	13 (9–17) 1–80
Total	3 (2–3) 1–22	3 (2–7) 1–25	11 (4–18) 1–69	17 (7–25) 1–89	5 (2–16) 1–89

Missing values: ^a^*n* = 1, ^b^*n* = 2, ^c^*n* = 5, ^d^*n* = 12, ^e^79 participants were discharged from Geriatric ward: pattern 1: *n* = 2, pattern 2: *n* = 18, pattern 3: *n* = 31, pattern 4: *n* = 28

**Table 2:** Characteristics of significant others

**3 months**	**Pattern 1** ***n*** **= 7**	**Pattern 2** ***n*** **= 28**	**Pattern 3** ***n*** **= 28**	**Pattern 4** ***n*** **= 26**	**Total** ***n*** **= 89**
Age, mean (SD), min-max	66 (14) 43–80	67 (11) 42–85	70 (14) 36–90	66 (14) 36–87	68 (13) 36–90
Sex, male, n (%)	2 (29)	4(6)	10 (36)	9 (35)	25 (28)
Relation					
Husband/wife, n (%)	4 (57)	23 (82)	21 (75)	19 (73)	67 (75)
Living apart, n (%)	1 (14)	1 (4)	1 (3.5)	–	3 (3)
Son/daughter, n (%)	2 (29)	4 (14)	5 (18)	6 (23)	17 (19)
Other, n (%)	–	–	1 (3.5)	1 (4)	2 (2)
Cohabiting, n (%)	4 (57)	23 (82)	23 (82)	20 (77)	70 (79)
**12 months**	***n*** **= 7**	***n*** **= 24**	***n*** **= 26**	***n*** **= 20**	***n*** **= 77**
Age, mean (SD), min-max	71 (10) 56–81	70 (10.9) 43–89	64 (15) 37–91	71 (11) 43–88	70 (12) 37–91
Sex, male, n (%)	0 (0)	3 (13)	10 (39)	5 (25)	18 (23)
Relation					
Husband/wife, n (%)	4 (57)	22 (91)	19 (73)	19 (95)	64 (83)
Living apart, n (%)	1 (14)	1 (4)	1 (4)		3 (4)
Son/daughter, n (%)	2 (29)	1 (4)	6 (24)	1 (5)	10 (13)
Cohabiting, n (%)	4 (57)	22 (92)	21 (81)	19 (95)	66 (86)

*SD* Standard deviation

The visits with the neurorehabilitation teams in relation to the stroke for which patients were included are presented in Table 3. In total, 1891 visits (team visits and individual provider visits) with the neurorehabilitation team were conducted, representing 14% of all outpatient contacts during the first year post-stroke. In pattern 2, 45% of the participants had their first visit with the neurorehabilitation team within 7 days of discharge, in comparison with 54% of participants in pattern 3, and 63% of participants in pattern 4. Visits were most frequent during the first quarter and decreased over the course of the year. The median total number of visits with the neurorehabilitation teams was in 1 visit for pattern 2 participants, 8 visits for pattern 3 participants, and 27 visits for pattern 4 participants. The majority (76%) of the neurorehabilitation team visits were conducted in the participants’ home”: 93% for pattern 2 participants, 89% for pattern 3 participants, and 70% for pattern 4 participants. Team visits (≥ 2 professions) accounted for 16% of visits, physiotherapists accounted for 36%, occupational therapists 24%, speech and language therapists 16%, social workers 8%, and dieticians 0.01%.

**Table 3:** Number of days to establish contact with the neurorehabilitation team and number of contacts with the neurorehabilitation team, in relation to the stroke for which they were included in the study, during the 12-month period, per pattern and total

	Pattern 2 ***n*** = 73			Pattern 3 ***n*** = 57			Pattern 4 ***n*** = 38		
	Persons, n (%)	Visits (n)	Median (IQR) min-max	Persons, n (%)	Visits (n)	Median (IQR) min-max	Persons, n (%)	Visits (n)	Median (IQR) min-max
**First visit**									
within 2 days	4 (5)			4 (7)			6 (16)		
between 3 and 7 days	29 (40)			27 (47)			18 (47)		
between 8 and 14 days	33 (45)			21 (37)			10 (26)		
≥ 15 days	7 (10)			5 (9)			4 (11)		
**Visits 1st quarter**									
Team visit									
At home	66	68	1 (0) 0–2	54	87	1 (1–2) 0–6	38	89	2 (1–3) 1–7
At outpatient clinic	3	3	0 (0) 0–1	3	4	0 (0) 0–2	7	18	0 (0) 0–5
Individual provider visit									
At home	27	38	0 (0–1) 0–3	51	295	4 (3–8) 0–12	37	515	12 (9–17) 0–38
At outpatient clinic	4	4	0 (0) 0–1	9	35	0 (0) 0–11	11	117	0 (0–3) 0–27
**Total 1st quarter**	**72**	**113**	**1 (1–2) 0–3**	**57**	**421**	**7 (5–10) 2–14**	**38**	**739**	**17 (14–24) 5–57**
**Visits 2nd quarter**									
Team visit									
At home	1	1	0 (0) 0–1	0	0	0 (0) 0–1	10	11	0 (0–1) 0–5
At outpatient clinic	0	0	0	3	3	0	6	18	0 (0) 0–2
Individual provider visit									
At home	0	0	0 (0) 0–0	14	27	0 (0) 0–7	29	216	4 (1–9) 0–33
At outpatient clinic	1	1	0 (0) 0–1	3	10	0 (0) 0–5	12	102	0 (0–1) 0–29
**Total 2nd quarter**	**2**	**2**	**0 (0) 0–1**	**19**	**40**	**0 (0–1) 0–7**	**36**	**347**	**7 (3–10) 0–46**
**Visits 3rd and 4th quarter**									
Team visit									
At home	0	0	0	0	0	0	7	11	0 (0) 0–3
At outpatient clinic	0	0	0	0	0	0	6	28	0 (0) 0–11
Individual provider visit									
At home	0	0	0 (0) 0–0	0	0	0 (0) 0–0	16	79	0 (0–2) 0–18
At outpatient clinic	0	0	0 (0) 0–0	0	0	0 (0) 0–0	6	111	0 (0) 0–75
**Total 3rd–4th quarter**	**0**	**0**	**0**	**0**	**0**	**0**	**20**	**229**	**1 (0–6) 0–76**
**Total 1st-4th quarter**	**73**	**115**	**1 (1–2) 1–3**	**57**	**461**	**8 (5–11) 4–15**	**38**	**1315**	**27 (20–34) 10–130**

*IQR* Interquartile range

**Fig. 2:**
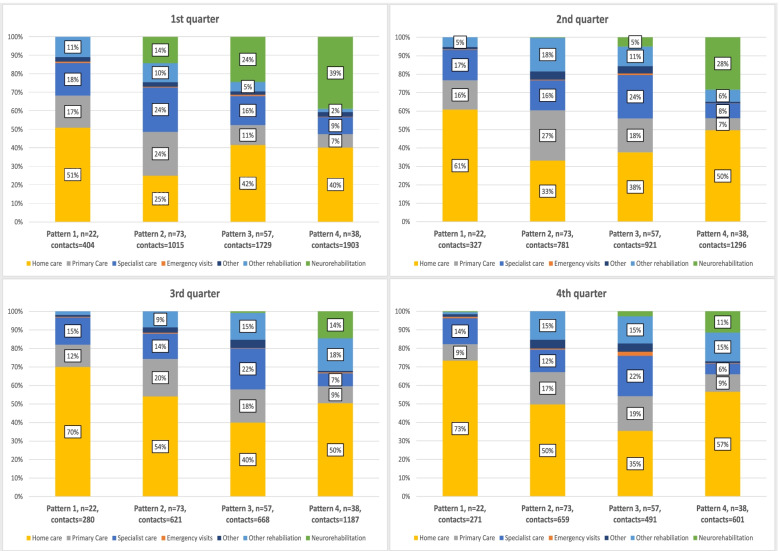
Total use of outpatient care, including visits with the neurorehabilitation team, during the first-year post-stroke

Total use of outpatient contacts, including visits with the neurorehabilitation team, is presented in Table 4 and Fig. 2. The median number of outpatient contacts differed between the patterns of healthcare use, pattern 1: 16.5 contacts; pattern 2: 20 contacts; pattern 3: 37 contacts; and pattern 4: 45 contacts. The total number of rehabilitation contacts accounted for 24% of the total use of outpatient contacts, and visits with the neurorehabilitation team accounted for 15% of the total use of outpatient contacts.

**Table 4:** Total outpatient care (*n* = 13,154 contacts), including visits with the neurorehabilitation team in relation to first and recurrent stroke, during the first year after stroke

	Pattern 1, ***n*** = 22		Pattern 2, ***n*** = 73		Pattern 3, ***n*** = 57		Pattern 4, ***n*** = 38		Total, ***n*** = 190	
Total outpatient contacts	Visits/users	Median (IQR) Min-Max	Visits/users	Median (IQR) Min-Max	Visits/users	Median (IQR) Min-Max	Visits/users	Median (IQR) Min-Max	Visits/users	Median (IQR) Min-Max
**Specialist care**	204/22	8 (4–10) 1–51	535/72	7 (3–11) 0–19	743/54	9 (4–15) 0–74	395/37	6.5 (5–12) 0–48	1877/185	7 (4–11) 0–74
**Emergency visits**	7/4	0 (0–0) 0–3	14/8	0 (0–0) 0–6	32/14	0 (0–1) 0–6	14/6	0 (0–0) 0–6	67/32	0 (0–0) 0–6
**Primary care**	180/21	5 (3–10) 0–53	695/69	5 (2–13) 0–62	567/52	6 (2–12) 0–70	390/37	6 (4–13) 0–41	1832/179	5.5 (2–12) 0–70
**Home care**	800/3	0 (0–0) 0–720	1177/14	0 (0–0) 0–657	1508/30	1 (0–41) 0–331	2347/19	0.5 (0–35) 0–670	5832/66	0 (0–17) 0–720
**Neurorehabilitation team**	1/1	0 (0–0) 0–1	146/73	1 (1–2) 1–23	484/57	8 (5–12) 4–20	1348/38	27.5 (21–36) 10–130	1979/169	4 (1–12) 0–130
**Other rehabilitation**^b^	69/10	0 (0–3) 0–27	400/40	1 (0–4) 0–70	354/44	2 (1–6) 0–66	419/29	3 (1–22) 0–39	1242/123	1 (0–6) 0–70
**Other**^a^	21/6	0 (0–1) 0–7	109/30	0 (0–1) 0–11	121/29	1 (0–2) 0–27	74/13	0 (2–2) 0–32	325/78	0 (0–2) 0–32

*IQR* Interquartile range. ^a^Includes visits such as laboratory testing, radiology and mammography. ^b^Included visits such as outpatient visits at primary care clinic not related to the neurorehabilitation team and specialized rehabilitation at a rehabilitation facility

The participants’ functioning at 3 and 12 months are presented in Table 5. The median IADL was 4 for patterns 1 and 2 and 1 for patterns 3 and 4. The same pattern was seen regarding BI, where it was found that patterns 1 and 2 had a lower level of disability compared to patterns 3 and 4.

**Table 5:** Participant functioning and caregiver burden on significant others at 3 and 12 months

Variable	Pattern 1	Pattern 2	Pattern 3	Pattern 4	Total
**3 months,** median (IQR) min-max	***N*** **= 20**	***N*** **= 63**	***N*** **= 45**	***N*** **= 36**	***N*** **= 164**
Barthel Index	100 (100–100) 80–100	100 (100–100) 65–100	100 (83–100) 30–100	95 (71–100) 20–100	100 (95–100) 20–100
KATZ					
PADL	6 (6–6) 5–6	6 (6–6) 3–6	6 (5–6) 2–6	6 (4–6) 0–6	6 (6–6) 0–6
IADL	4 (3–4) 0–4	4 (2–4) 0–4	1 (0–4) 0–4	1 (0–2) 0–4	3 (1–4) 0–4
SIS Recovery	90 (63–97) 30–100	85 (70–98) 0–100	70 (50–80) 0–100	53 (31–77) 0–100	75 (50–90) 0–100
Walking ability, n (%)					
Walks independently without aid and support	18 (90)	51 (81)	30 (67)	24 (67)	123 (76)
Walks with walking aid	1 (5)	8 (13)	11 (24)	5 (14)	25 (15)
Unable to walk/walks with assistance and support	1 (5)	4 (6)	3 (7)	7 (19)	15 (9)
**Number of significant others**	***N*** **= 7**	***N*** **= 28**	***N*** **= 28**	***N*** **= 26**	***N*** **= 89**
Caregiver Burden Scale	41 (27–44) 24–48	30 (23–40) 22–76	38 (28–49) 23–69	41 (32–52) 22–72	36 (28–48) 22–76
**12 months,** median (IQR) min-max	***N*** **= 20**	***N*** **= 53**	***N*** **= 41**	***N*** **= 32**	***N*** **= 146**
Barthel Index	100 (96–100) 75–100	100 (100–100) 50–100	100 (90–100) 5–100	95 (76–100) 30–100	100 (95–100) 5–100
KATZ					
PADL	6 (6–6) 5–6	6 (6–6) 1–6	6 (5–6) 0–6	6 (5–6) 1–6	6 (6–6) 0–6
IADL	4 (2–4) 0–4	4 (2–4) 0–4	3 (1–4) 0–4	2 (0–4) 0–4	3 (1–4) 0–4
SIS Recovery	90 (80–100) 5–100	90 (80–98) 50–100	70 (43–90) 0–100	60 (42–79) 0–100	80 (60–92) 0–100
Walking ability, n (%)					
Walks independently without aid and support	17 (85)	43 (81)	26 (63)	24 (75)	110 (78)
Walks with walking aid	0	6 (11)	7 (17)	2 (6)	15 (11)
Unable to walk/walks with assistance and support	1 (5)	1 (3)	7 (17)	6 (19)	15 (11)
Montreal Cognitive Assessment	27 (6) 15-30^a^	27 (4) 10-30^b^	23.5 (7) 9-30^c,d^	21 (7) 8-29^d^	25 (7) 8-30^e^
**Number of significant others**	***N*** **= 7**	***N*** **= 24**	***N*** **= 26**	***N*** **= 20**	***N*** **= 77**
Caregiver Burden Scale	31 (26–55) 23–60	27 (22–33) 21–58	37 (27–54) 22–67	44 (30–63) 23–71	33 (26–53) 21–71

*IQR* Interquartile range, *PADL* Personal activities of daily living, *IADL* Instrumental activities of daily living. Missing values: ^a^*n* = 7, ^b^*n* = 27, ^c^*n* = 25, ^d^*n* = 12, ^e^*n* = 71

All patterns increased their median self-perceived recovery from stroke between 3 and 12 months except for pattern 1.

At 3 months, the median caregiver burden was 41 for patterns 1 and 4, 29.5 for pattern 2, and 38 for pattern 3. At 12 months, the burden had decreased in pattern 1 from a median of 41 to 31, while for pattern 4 it had increased from a median of 41 to 43.5.

From the multivariable linear regression calculated to explore associations between the independent variables and the total number of visits with the neurorehabilitation team, a significant regression equation was found: (F(5,170) = 18,905, *p* < .000), with an R-square of .357. The final model, as seen in Table 6, showed that female sex and longer length of stay were associated with a higher number of visits with the neurorehabilitation team. Living alone, a higher self-rated recovery and being able to walk independently were associated with a lower number of contacts with the neurorehabilitation team.

**Table 6:** Final multiple linear regression model of associations between independent variables and total visits with the neurorehabilitation team

Total visits with the neurorehabilitation team						
Independent factors	Unstandardized coefficients				***P***-value	Standardized
	β	95% CI				B
Constant	0.929	0.730 - -1.128			0.000	
Female	0.177	0.041 - -0.314			0.011	0.172
Living alone	−0.201	−0.342 - -0.060			0.006	−0.190
Length of stay	0.014	0.008–0.020			0.000	0.333
Self-rated recovery	−0.003	−0.005 - -0.001			0.014	−0.160
Walks independently without aid or support	−0.270	−0.416	–	−0.124	0.000	−0.268

*CI* Confidence interval

From the multivariable linear regression calculated to explore associations between the independent variables and total number of outpatient contacts (dependent), a significant regression equation was found: (F(4,171) = 16,444, *p* < .000), with an R-square of .278. The final model, seen in Table 7, showed that female sex, receiving home help services before stroke, a longer length of stay, and higher comorbidity were associated with a higher number of outpatient contacts.

**Table 7:** Final multiple linear regression model of associations between independent variables and the total outpatient contacts

Total outpatient contacts				
Independent factors	Unstandardized coefficients		***P***-value	Standardized
	β	95% CI		B
Constant	2.760	2.544–2.976	0.000	
Female	0.319	0.045–0.593	0.023	0.152
Home help services before stroke	0.620	0.282–0.959	0.000	0.239
Length of stay	0.029	0.017–0.040	0.000	0.340
Comorbidity	0.109	0.003–0.202	0.044	0.138

*CI* Confidence interval

## Discussion

This is the first study to explore and describe in detail healthcare use during a one-year period post-stroke, and health outcomes at 3 and 12 months, for patients receiving RBT to subsequent rehabilitation in the home.

All the participants in the present study received a referral to continued rehabilitation in the home environment with the neurorehabilitation team, however, the amount of rehabilitation utilized differed between the participants. Pattern 1, representing 12% of the sample, had no visits with the neurorehabilitation team, despite being similar to pattern 2 (those receiving 1–3 visits) in terms of patient characteristics, disease-related data, and functioning at discharge. The majority of participants in pattern 1 declined a visit from the neurorehabilitation team. Referral-based transitions have been reported to result in safety incidents such as insufficient information, incomplete referrals, and delayed processes in transition from the hospital to the community [28, 29]. It is conceivable that follow-up with a neurorehabilitation team might assist in navigating the healthcare system and compensate for a lack of information. Although participants in pattern 1 did not access the neurorehabilitation teams, their overall low healthcare use during the year and their high level of functioning indicates that these participants’ decision to decline services was reasonable. Nevertheless, the high caregiver burden at 3 months in pattern 1 similar to that for participants in pattern 4, who had a higher level of disability may indicate that significant others experienced a need for support due to their sudden new responsibility, which might impact their own life situation. Being a significant other to a person with stroke often means an increase in pressure and responsibility [30]; however, significant others who have contact with a multidisciplinary team report a lower caregiver burden [31]. Our results are in line with previous studies indicating that interventions directed primarily at patients with stroke may have favorable effects on their significant others [32].

For all three patterns who had at least one visit from the neurorehabilitation team, around 50% had their first visit within 1 week, and ≥ 90% had their first visit within 14 days. Further, our results indicate that there seems to be a flexibility and priority in the RBT as people with a more severe stroke appeared to receive the first visit sooner than those with a milder stroke. The opportunity for early follow-up is important, since coming home after discharge can result in feelings of abandonment, insecurity about post-discharge procedures, and lack of support for self-management after discharge [7, 33]. The visits with the neurorehabilitation team and other outpatient contacts were most frequent during the first quarter and decreased over the course of the year, a finding in line with previous research [34]. One reason for the decrease in total outpatient contacts could be aligned with a higher self-perceived recovery. Although all patterns, except pattern 1, showed gains in self-perceived recovery, it may differ across patterns. As self-perceived recovery is likely to be impacted by many individual factors, this needs further exploration in future studies, including potential variation over time. The decrease in visits with the neurorehabilitation teams after 6 months, together with increased use in other rehabilitation services as shown in pattern 4, might suggest a continued need for rehabilitation after the contact with the neurorehabilitation team has ended. Unmet rehabilitation needs have previously been reported in a similar context and time-span [35]. Hence, further investigation is warranted to explore whether services provided after 6 months meets the actual needs of patients with stroke and aligns with current recommendation and guidelines for stroke care and rehabilitation. The majority of the visits with the neurorehabilitation team were conducted in the participants’ homes, which is in line with recommendations for post-discharge rehabilitation for people with mild to moderate stroke [17]. A similar pattern of contact with home rehabilitation was reported in a study with a comparable organization of home-based rehabilitation [36]. One implication may be that this type of care trajectory offers a flexible organization for patients with stroke, both for individual assessments followed by shorter interventions, and for long-term support in the adaptation to their new life situation.

In the present study, being female and having had a longer hospital stay were associated with a larger number of visits with the neurorehabilitation team, while living alone, having a higher self-reported recovery and being able to walk independently were associated with a lower number of visits with the neurorehabilitation team. These results appear to mirror the variation in characteristics between the four different patterns i.e., sex, self-reported recovery, and walking ability, while the proportion of people living alone did not differ among the patterns. The association between the ability to walk independently and lower resource use is in line with previous studies [37, 38]. The unexpected association between living alone and a lower number of contacts with the neurorehabilitation team needs to be studied further. One possible explanation might be that living together with someone could facilitate access to healthcare services and support for taking part in rehabilitation.

Plausible common reasons for higher use of healthcare in women, such as stroke severity, age, and comorbidity [39], do not appear to explain the higher number of contacts with the neurorehabilitation team found in the present study. The reasons for these differences must therefore be sought elsewhere. Women have been reported to utilize more healthcare compared to men [40, 41]. Women have also been described as being less likely to achieve independence in ADL [42, 43] and hence might be in greater need of rehabilitation and other healthcare services. Furthermore, women are more likely to prefer rehabilitation in the home environment than men are [44], which might be another reason for the association between gender and the number of neurorehabilitation visits.

For total outpatient contacts, being female, having received help from home services before stroke, having had a longer hospital stay, and higher comorbidity were associated with higher use. These results are in line with previous studies that have used the CCI to assess and categorize comorbidity; a higher score on that index has been shown to predict higher healthcare costs [45, 46].

Visits with the neurorehabilitation team accounted for only 15% of the total number of outpatient contacts. Combining the visits with the neurorehabilitation team and other types of rehabilitation, total rehabilitation contacts accounted for 24% of the total amount of outpatient contacts.

There is a lack of systematic investigations to understand patients’ journeys across care transitions and the stroke continuum, as well as studies exploring existing services and their contents. Furthermore, because there is a large variation in the organization of healthcare systems, comparisons are hard to make. However, two recent studies in Ontario, Canada, investigated stroke care trajectories and referral patterns in stroke care and rehabilitation [16, 47]. Similar to our findings, Janzen et al. found that receiving a referral to outpatient stroke rehabilitation did not automatically mean that all participants actually received rehabilitation services after discharge [16]. Hall et al. described the post-stroke trajectory as consisting of several different trajectories that differed in terms of their adherence to best stroke care practices [47]. Further investigation is merited on whether the patterns of healthcare use found in this study adhere to best stroke care practices [5] and meet the needs of the patients and significant others.

The findings of the present study must be interpreted with caution due to the small sample size and the large proportion of participants with very mild or mild stroke. Nevertheless, participants with severe stroke were represented in all patterns. We chose to identify patterns based on number of visits and time for expected recovery of impairments i.e., 6 months [15]. We acknowledge that patterns of healthcare use can be based on different points of departure and the distinction between those with 1–3 visit and those with 4 or more visits can be questioned. However, based on our clinical experience we considered 1–3 visits as follow-ups and identified them as a separate group. The recruitment of participants from two different hospitals might have influenced the results of the different patterns. As our sample size was small and both hospitals are situated within the same region, with the same clinical guidelines and opportunities for continued rehabilitation and services post discharge, we chose to analyse the sample as a whole. It would be interesting to explore potential regional differences of healthcare use, however that would require a larger study sample. One strength of the present study is its use of data from the regional database regarding healthcare use, which eliminates the risk of recall bias. Further strengths are the prospective study design and collection of data through face-to-face interviews using standardized questionnaires, representing both performance-based, self-assessed, and patient-reported outcomes. The detailed description further contributes to an increased understanding of the varying needs and use of healthcare during the first year after stroke. However, the content of each visit in relation to patients’ needs and current evidence-based practice remains to be explored.

## Conclusions

There was a large variation in the number of visits participants had with the neurorehabilitation team and other outpatient contacts. The results of this study indicate that there is no generic pattern of healthcare use during the first-year post-stroke in patients receiving RBT to continued rehabilitation in the home. The different patterns of healthcare use seemed to reflect the participants’ level of functioning. However, there is a need to further investigate how follow-up and rehabilitation correspond to evidence-based practice and the needs of patients and their significant others from a short- and long-term perspective. 

## Data Availability

The datasets generated and/or analysed during the current study are not publicly available but can be available upon reasonable request. As data can indirectly be traced back to the study participants, according to the Swedish and EU personal data sharing legislation, access can only be granted upon request. Request for access to the data can be put to our Research Data Office (rdo@ki.se) at Karolinska Institutet and will be handled according to the relevant legislation. In most cases, this will require a data processing agreement or similar with the recipient of the data.

## Abbreviations

ESD: Early supported discharge
RBT: Referral-based transition
BI: Barthel Index
ADL: Activities of daily living
CCI: Charlson Comorbidity Index
PADL: Personal activities of daily living
IADL: Instrumental activities of daily living
CBS: Caregiver Burden Scale
IQR: Interquartile range
SD: Standard deviation
CI: Confidence interval
